# The Endothelial‐Independent Effect of Desmopressin, In Vitro, on Platelet Function

**DOI:** 10.1155/ah/9928082

**Published:** 2025-10-19

**Authors:** Michael V. Forner, Etheresia Pretorius, Chantelle Venter, Sean Chetty

**Affiliations:** ^1^ Department of Anesthesiology and Critical Care, Faculty of Medicine and Health Sciences, Stellenbosch University, Cape Town, Western Cape, South Africa, sun.ac.za; ^2^ Department of Physiological Sciences, Faculty of Sciences, Stellenbosch University, Cape Town, Western Cape, South Africa, sun.ac.za

**Keywords:** desmopressin, microclots, platelet function, platelets, viscoelastic testing

## Abstract

Platelets form the nidus around which the primary hemostatic cascade is amplified and propagated. Desmopressin (DDAVP) has been shown to enhance platelet function through interaction with the endothelium. Evidence suggests that an alternative mechanism of action may exist. We aimed to determine the effect of DDAVP on platelet function and microclotting of plasma proteins in samples from healthy volunteers. We analyzed blood samples in 20 healthy volunteers with no coagulation abnormalities. Control and test samples were drawn from each participant. DDAVP was added in vitro to test samples. All samples were subjected to PFA‐200, viscoelastic (VET) and fluorescence microscopy testing. DDAVP increased TEG MA and decreased K‐time in experimental samples. There was no difference between the means in the clotting times in either of the PFA‐200 groups. Fluorescence microscopy examining platelet activation and microclot formation showed significant increases in both parameters in the test samples. The results contrast current literature, which suggests that DDAVP has no effect on platelet function independent of the endothelium. This is the first study demonstrating an in vitro effect of DDAVP on platelets examined by VET and microscopy. We conclude that a relationship exists between DDAVP exposure, in vitro, platelet activation and microclot formation.

## 1. Introduction

Platelets form the nidus around which the primary hemostatic cascade is amplified and propagated [1]. Desmopressin (DDAVP), an analog of antidiuretic hormone (ADH), has been shown to enhance platelet function through interaction with the vascular endothelium [2]. First used in 1977, this mechanism of action has been exploited for nearly 50 years in the management of bleeding patients with Hemophilia A and von Willebrand’s disease (vWD) [3–5].

Since the 1980s, there has been a debate as to whether this is the sole mechanism by which DDAVP interacts with platelets. In vitro and in vivo evidence suggests that an alternative mechanism of action may also be in effect [6, 7]. ADH, the naturally occurring neurohypophysial hormone is known to activate platelets through the V1‐receptor. Yang et al. demonstrated that platelets preincubated with DDAVP competed with ADH for the V1‐receptor [8]. Prior to this study, it was believed that DDAVP was V2‐receptor selective, and although the study did not support the notion that DDAVP was able to activate the platelets directly, it highlighted the fact that DDAVP interacts with platelets through mechanisms not fully elucidated.

In 2014, Colucci et al. investigated the effect of DDAVP on platelet function, in vivo, in patients with congenital mild platelet dysfunction [2]. They concluded that some of DDAVP’s effects on platelet aggregation are mediated through increases in von Willebrand factor (vWF) concentration, which in turn enhances agonist‐induced platelet aggregation. Their work did not support a direct effect of DDAVP on platelet aggregation; however, it did suggest a role for alternative mechanisms of platelet activation involving activation of sodium pumps and sustained increases in intracellular calcium concentration [2]. These findings are supported by an earlier study conducted in 2004 by Tomasiak et al., who showed that the DDAVP‐mediated procoagulant effects on platelets were mitigated by 5‐(N‐ethyl‐N‐isopropyl) amiloride (EIPA), a sodium‐hydrogen exchange pump inhibitor [9].

Glycoprotein (GP) IIb/IIIa plays an essential role in platelet–platelet aggregation and binding of platelets to the vascular endothelium, as it provides the binding sites for both vWF and fibrinogen [10]. Reiter et al. investigated the effect of DDAVP on healthy individuals treated with a GP IIb/IIIa inhibitor. They showed that DDAVP increased the rate of normalization of in vitro platelet dysfunction induced by GP IIb/IIIa inhibitors [11]. Considering the current accepted mechanism of DDAVP‐induced procoagulant effects that is mediated through increased levels of vWF, this study provides further evidence that the effect of DDAVP on platelet function, nor its complete mechanism of action, is not fully understood. This assertion is further supported by Cattaneo et al., who showed that DDAVP further corrected bleeding times in patients with severe vWD in whom levels of vWF were already corrected to normal concentrations by cryoprecipitate infusion [7].

We therefore aimed to determine the effect of DDAVP on platelet function, in vitro, in samples from healthy volunteers.

## 2. Materials and Methods

### 2.1. Study Design

This cross‐sectional analytical, laboratory‐based in vitro study analyzed blood samples of 20 healthy volunteers. Ethical approval for the study was granted by the Human Research Ethics Committee, Stellenbosch University, South Africa (Reference number: S22/08/140). The study was conducted in accordance with the principles of Good Clinical Practice and with the Declaration of Helsinki. The study was conducted between June and July 2023.

### 2.2. Study Population

Twenty healthy, nonpregnant volunteers between the ages of 18 and 50 years old were recruited by convenience sampling for participation in the study. Participants were screened by the primary investigator (PI) for eligibility to participate in the study, according to the following criteria:

Inclusion criteria:

• Healthy volunteers 18–50 years old of any ethnicity.

Exclusion criteria:

• Thrombocytopenia (platelet count of < 150 × 10^9^ L^−1^).
• Thrombocytosis (platelet count of > 450 × 10^9^ L^−1^).
• Organ failure (controlled or uncontrolled).
• Elevated serum urea, i.e., > 7.1 mmol/L.
• Currently taking any form of prescribed or over‐the‐counter anticoagulant or antiplatelet therapy including but not limited to:
  ◦ Cyclooxygenase enzyme inhibitors
  ◦ P2Y12 receptor antagonists
  ◦ Phosphodiesterase inhibitors
  ◦ GP IIb/IIIa receptor antagonists
  ◦ Protease‐activated receptor (PAR) antagonists
  ◦ Warfarin
  ◦ Heparin
  ◦ Direct thrombin inhibitors
  ◦ Factor Xa inhibitors
  ◦ Prostaglandin analogs
• Hereditary or newly acquired bleeding diathesis including but not limited to disorders of coagulation (Hemophilia A/B, Factor V Leiden, Protein C and S deficiency, antiphospholipid syndrome etc.) as well as disorders of platelet function (vWD, Bernard–Soulier syndrome, and Glanzmann’s thrombasthenia).
• Anemia or erythrocytosis as defined by laboratory‐specific ranges for age, sex, and ethnicity.
• Hematocrit < 35% or > 55%.
• Acute illness within 10 days of blood sampling.

Informed consent was obtained from all participants.

## 3. Sample Size

A minimum sample size of 19 participants was calculated to adequately power (0.8) the study with an alpha of < 0.05. The statistical software used was Stata, Version 17 (StataCorp LLC, Texas).

## 4. Data Collection

Screening blood samples were obtained using a 21G BD Eclipse needle and BD Vacutainer one‐use holder. Whole blood samples were obtained for the screening blood tests: hemoglobin concentration, platelet count, prothrombin time/international normalized ratio, activated partial thromboplastin time, and urea. The first 20 participants who met the inclusion and exclusion criteria were included in the study.

Eligible participants were given an information document (available on request) on specific foods and medications to abstain from for the next 10 days. On Day 11, blood samples were collected into six 4.5 mL 3.2% (0.105 M) sodium citrate buffered tubes (citrate tubes), one 3.0 mL whole blood tube with spray‐coated K2EDTA, and one 4.0 mL SST tube with silica clot activator, polymer gel, and silicone‐coated interior.

Participant samples were analyzed using thromboelastography (TEG6s, Haemonetics Corporation, Boston, USA) and platelet function analyzer (PFA‐200 System, Siemens Ltd., Johannesburg, South Africa). The blood samples were also analyzed using fluorescence microscopy.

### 4.1. Sample Preparation

Three citrate tubes were processed as controls. One tube for TEG analysis and two for PFA‐200 and fluorescence microscopy (platelet analysis and microclot analysis). DDAVP (Ferring Pharmaceutical; South Africa) 5 μL (4 μg/mL intravenous ampoule) was added to the remaining three citrate tubes. The same analyses were run on the DDAVP test samples as for the controls. Samples were processed after 30 min but within 4 h of phlebotomy and kept at room temperature.

The INNOVANCE PFA‐200 system is composed of two separate tests performed using two individual cartridges: the collagen/epinephrine (CEPI) and collagen/adenosine diphosphate (CADP) cartridges. Both tests were performed on each of the control and test samples. The tests were carried out by pipetting a sample of citrated whole blood into the CEPI and CADP cartridges which contain a reservoir, capillary and aperture lined with a membrane impregnated with the designated agonists. Aperture closure time is documented when flow through the aperture has stopped due to platelet aggregation [12].

As opposed to the older generation TEG 5000 which used a cup, to which a whole blood sample and reagent were added, the TEG6s uses disposable cartridges (Global Hemostasis Cartridge). The cartridge is manufactured with four separate channels primed with four different assays: Kaolin TEG, Kaolin TEG with heparinase, Rapid TEG, and TEG Functional fibrinogen [13].

Citrated whole blood (control and test samples) was pipetted using the manufacturer‐provided transfer pipette to the cartridge sample port. The cartridge was then inserted into the TEG6s Hemostasis analyzer and allowed to run to completion. Data were collected from the results of the Kaolin TEG.

#### 4.1.1. Preparation of Platelet‐Poor Plasma (PPP) and Platelets in the Hematocrit Fraction

One control and one test citrate sample were prepared per participant. PPP was prepared using a centrifugation step of 15 min at 3000 RPM. These microscopy preparation methods have previously been discussed in various papers [14, 15]. The hematocrit fraction of each sample was used to study platelet fragility. Unlike traditional methods that involve the study of platelets in whole blood, buffy coat, or platelet‐rich plasma with the addition of stabilizers to prevent activation, our approach involves spinning whole blood to create PPP and studying the platelets in the remaining hematocrit fraction. We intentionally avoid adding platelet stabilizers, as our goal is to compare the fragility of platelets between treated and untreated samples. The use of stabilizers or inhibitors (e.g, indomethacin, PGE1, ASS, or apyrase) could interfere with the effects of DDAVP that we add to the samples, potentially confounding our results. The untreated sample, which also underwent centrifugation, serves as our control for assessing platelet activation due to the centrifugation process. This ensures that any observed effects can be specifically attributed to DDAVP.

#### 4.1.2. Analysis of Microclots in PPP

The PPP fraction contains clots of fibrin(ogen) and plasma proteins, otherwise known as microclots. These microclots are also present in small quantities in healthy samples [16]. To view microclots in the PPP, before and after exposure to DDAVP, thioflavin T (ThT) (exposure concentration: 5 μM) (Sigma‐Aldrich, St. Louis, MO, USA) was added to the PPP and incubated for 30 min. After placing a 3 μL drop of the sample on a microscope slide, the sample was viewed with a Zeiss Axio Observer 7 fluorescence microscope with a Plan‐Apochromat 63 ×/1.4 Oil DIC M27 objective (Carl Zeiss Microscopy, Munich, Germany) using the excitation wavelength of 450–488 nm and emission from 499 to 529 nm.

#### 4.1.3. Analysis of Hematocrit Platelets

After preparing a hematocrit sample and removing the PPP, two fluorescent markers, CD62P (for P‐selectin) (IM1759U, Beckman Coulter, Brea, CA, USA) and PAC‐1 (for activated GP IIb/IIIa) (340,507, BD Biosciences, San Jose, CA, USA) were added to the hematocrit. CD62P/P‐selectin is released from the cellular granules during platelet activation and then moves to the surface of the platelet membrane. The antibody PAC‐1 detects the neoepitope of active GP IIb/IIIa. PAC‐1 antibody binding is correlated with platelet activation. PAC‐1 and CD62P/P‐selectin markers were applied to both the untreated (control) and the DDAVP‐exposed samples in each of the 20 pairs. The PAC‐1 and CD62P/P‐selectin signals from the untreated (control) samples served as the baseline for assessing platelet activation in the corresponding DDAVP‐exposed samples. After the samples were incubated at room temperature for 30 min, the samples were also viewed using the Zeiss Axio Observer 7 fluorescence microscope with a Plan‐Apochromat 63×/1.4 Oil DIC M27 objective (Carl Zeiss Microscopy, Munich, Germany). We used excitation wavelength 406–440 nm and the emission at 546–564 nm (PAC‐1), and excitation 494–528 nm and the emission at 618–756 nm (CD62P) [15].

### 4.2. Data Analysis

Stata, Version 17 (StataCorp LLC, Texas) was used for the PFA‐200 and TEG data analyses. Microclot and platelet activation scores were statistically analyzed using GraphPad 10.3. Categorical data were presented as frequencies and percentages. The Shapiro–Wilk test was used to assess data distribution. Quantitative data collected from the TEG (R‐time, K‐time, angle, and MA) and PFA‐200 were parametric, continuous data, with two paired groups, and therefore, analyzed with a paired *t*‐test. Quantitative data from the fluorescence microscopy and TEGLY30 were nonparametric paired data and were analyzed with the Wilcoxon signed‐rank test. The differences/effects are reported as the mean difference with a 95% confidence interval derived. Significance (*α*) was set at 5%. Data files and micrographs are available on request.

#### 4.2.1. A Grading System for Plasma Microclot Formation and Platelet Activation, Spreading and Clumping

We used a platelet and microclot grading system previously developed by Laubscher et al. [17]. We also used their grading system for assessing microclot presence and platelet activation, as shown in Tables 1 and 2. Together with TEG analysis, this grading could give insights into clotting pathology.

**Table 1:** Microclot criteria to determine the amount of microclots in a platelet‐poor plasma (PPP) sample.

Score	Presence of microclots in platelet‐poor plasma
1	Very few areas of plasma protein misfolding (≤ 1 μm) are visible with a few, ≤ 10 μm, microclots
2	Very few areas of plasma protein misfolding (≤ 1 μm) are visible with scattered/mild, ≤ 10 μm, microclots
3	Moderate areas of plasma protein misfolding are visible as microclots ≥ 15 µm
4	Severe areas of plasma protein misfolding are visible as large microclots

*Note:* Previously published in Laubscher et al. [17].

**Table 2:** Platelet activation criteria showing the level of spreading, as well as clumping of platelets in the hematocrit sample.

Score	Spreading	Score	Clumping
1	Activation with pseudopodia	1	None
2	Mild	2	Mild
3	Moderate	3	Moderate
4	Severe	4	Severe

*Note:* Previously published in Laubscher et al. [17].

## 5. Results

We tested blood samples from 20 healthy volunteers, 11 males and 9 females. Baseline population characteristics and screening results are shown in Table 3.

**Table 3:** Population characteristics and laboratory screening results.

*n* = 20	Median (IQR)
Age (years)	32.50 (31.00–35.50)
Hemoglobin (g/dL)	14.15 (13.50–14.7)
Platelets (× 10^9^/L)	313.50 (253.50–337.50)
Urea (mmol/L)	4.95 (4.30–5.40)
aPTT (s)	25.2 (23.95–26.05)
PT (s)	10.3 (10.15–10.75)
INR	1.00 (1.00–1.00)

Abbreviations: aPTT, activated partial thromboplastin time; INR, international normalized ratio; IQR, interquartile range; PT, prothrombin time.

### 5.1. PFA‐200

**Figure 1:**
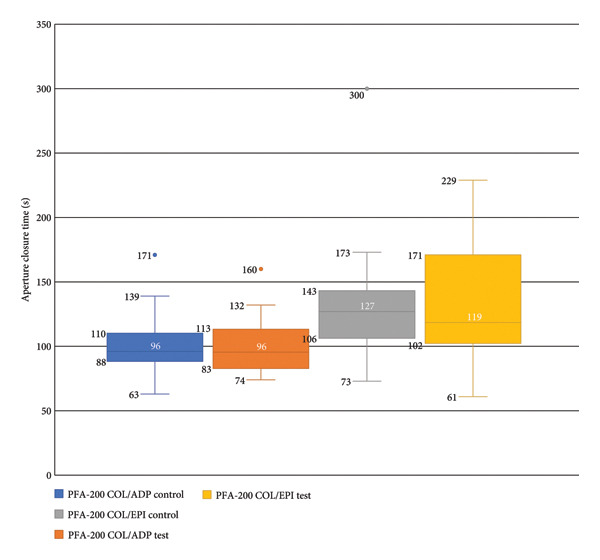
Box plot comparing the results of the PFA‐200 tests in the control and DDAVP test samples for both the collagen/ADP and collagen/epinephrine cartridges. Center lines represent median values; boxes include the interquartile range (IQR). Whiskers include 1.5 times the IQR. Outliers are shown as individual data points.

Platelet function according to the PFA‐200 tests showed no statistically significant difference between control and test sample means of the CADP and CEPI tests. The difference between the means was 0.2 s (CI −5.22 to 5.62, *p* = 0.939) and 0.0 s (CI −19.3 to 19.3, *p* = 1.0) respectively (Figure 1).

### 5.2. VET Testing (TEG6s)

Thromboelastography showed a statistically significant reduction of 0.11 min (95% CI: 0.019–0.21) in the K‐time test sample mean (1.49 min ± 0.26) versus control samples (mean 1.61 min ± 0.25), *p* value 0.02. An increase in the DDAVP sample’s MA of 0.555 mm (95% CI: 0.046–1.06), *p* = 0.034, was also noted. No difference between the means of the control and test samples was noted in the R‐time or angle (Table 4).

**Table 4:** Paired *t*‐test results of TEG6s control versus DDAVP.

*n* = 20	Control $\bar{x}$ (±SD)	DDAVP $\bar{x}$ (±SD)	DDAVP $\bar{x}$‐control $\bar{x}$	95% CI	*p* value
R‐time (min)	6.240 (±0.86)	6.130 (±0.81)	−0.110	0.280 to −0.500	0.562
K‐time (min)	1.650 (±0.25)	1.490 (±0.26)	−0.115	−0.019 to −0.210	0.020
Angle (°)	70,210 (±2.35)	71,075 (±2.65)	0.865	1845 to −0.115	0.080
MA (mm)	56,645 (±3.80)	57,200 (±3.56)	0.555	1064 to 0,46	0.034

*Note:* A Wilcoxon signed‐rank test of the TEGLY30 data revealed no statistically significant difference between the control (median: 0.8%) and DDAVP (median: 0.8%) groups, *p* = 0.59. *n*, number of participants; $\overset{¯}{x}$, mean.

Abbreviations: CI, confidence interval; SD, standard deviation.

Figures 2 and 3 show platelets and PPP before and after exposure to DDAVP. We also scored the microclot and platelet activation using our microclot and platelet grading system [17]. Significant microclot formation was present after exposure to DDAVP (*p* ≤ 0.0001). Significant platelet spreading was noted (*p* = 0.003), but no significant platelet clumping was found (*p* = 0.2).

Figure 2Platelet activation in the hematocrit, before and after exposure to DDAVP. (a), (c), and (e) show representative micrographs of the three participants before exposure, while (b), (d), and (f) are representative micrographs of the same three participants, after their blood samples were exposed to DDAVP showing hyperactivation. The arrow in (f) indicates the platelet clumping.

**Figure:**
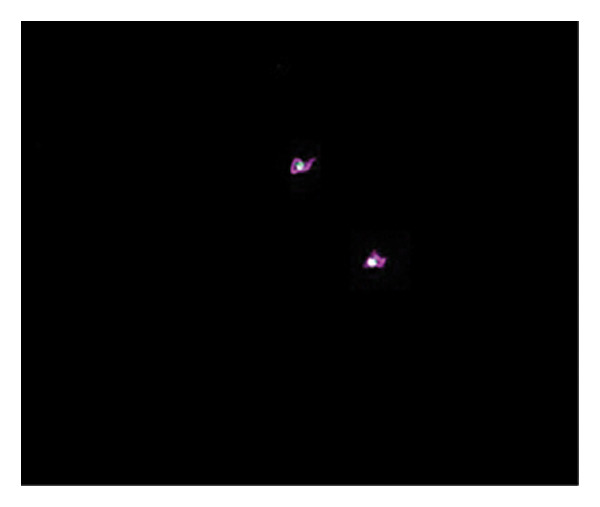
(a)

**Figure:**
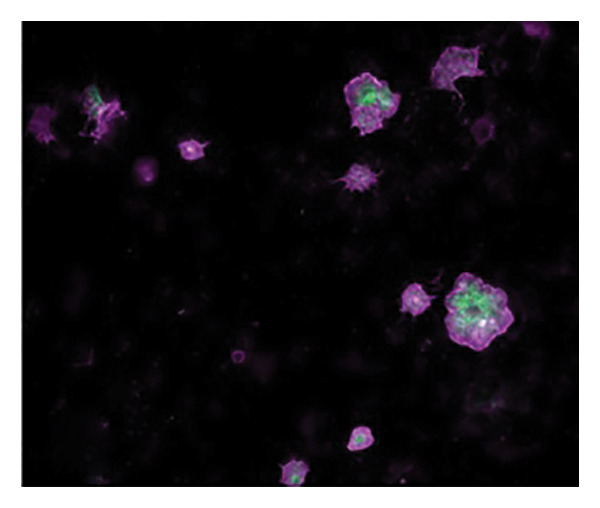
(b)

**Figure:**
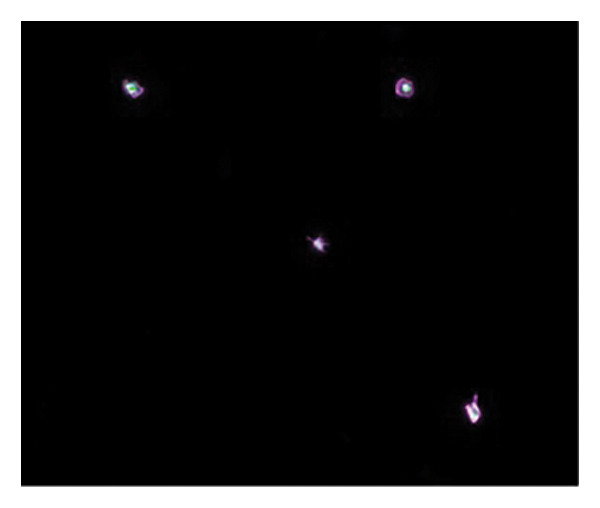
(c)

**Figure:**
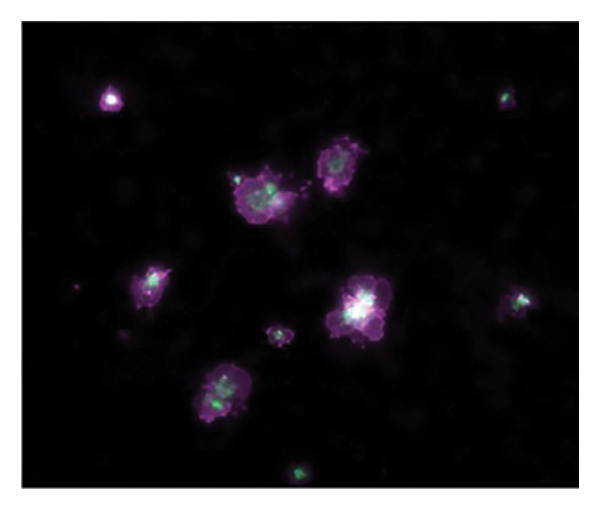
(d)

**Figure:**
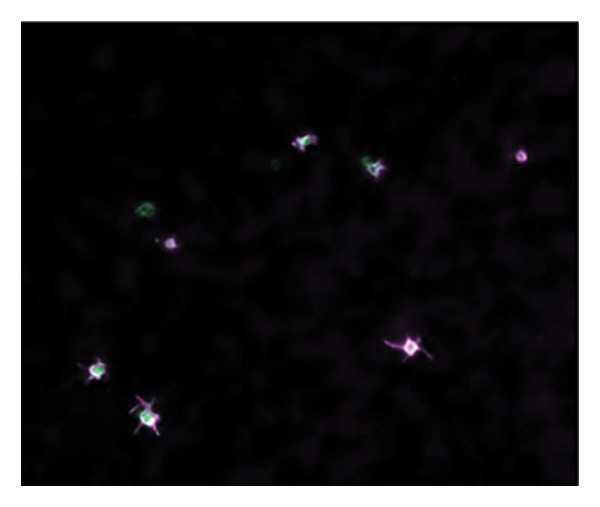
(e)

**Figure:**
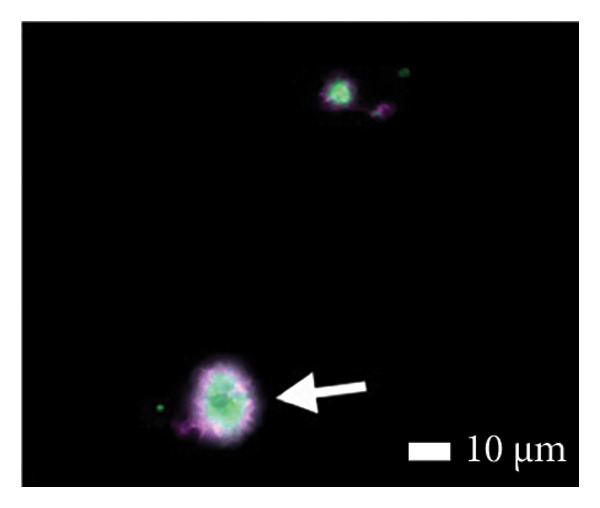
(f)

Figure 3Microclot presence in platelet‐poor plasma (PPP) before and after exposure to DDAVP. (a), (c), and (e) show representative micrographs of three participants before exposure, while (b), (d), and (f) are representative micrographs of the same three participants, after their blood samples were exposed to DDAVP.

**Figure:**
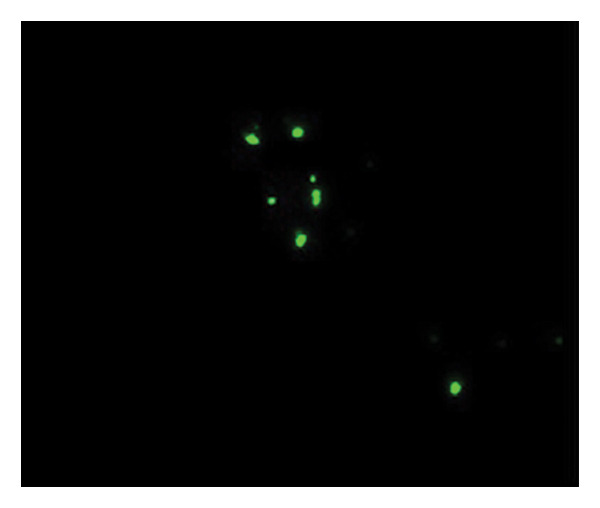
(a)

**Figure:**
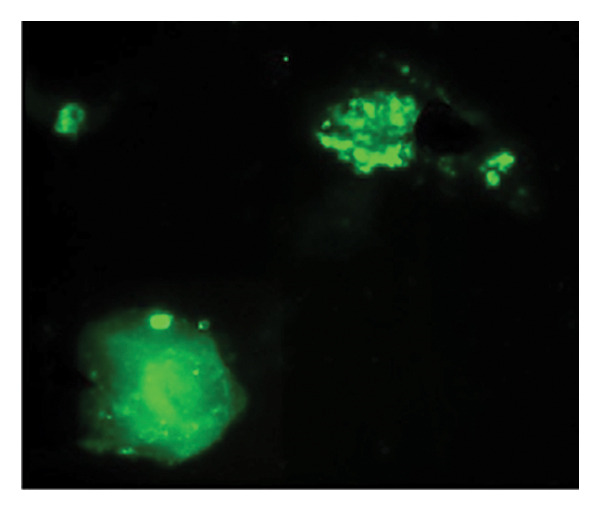
(b)

**Figure:**
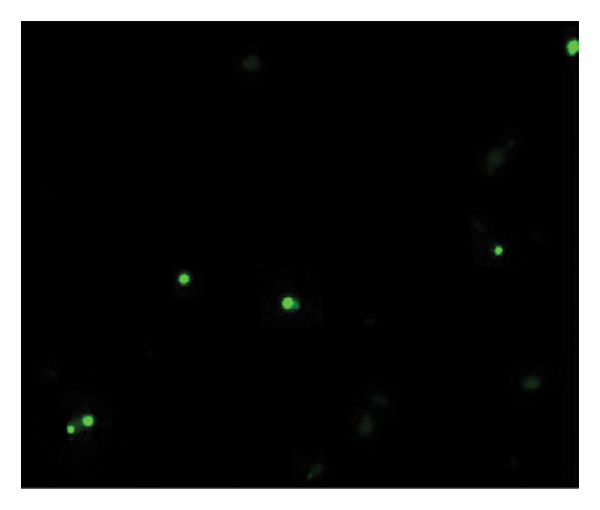
(c)

**Figure:**
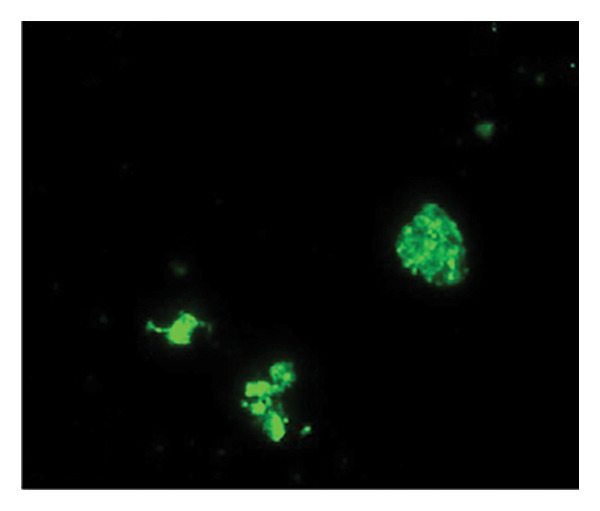
(d)

**Figure:**
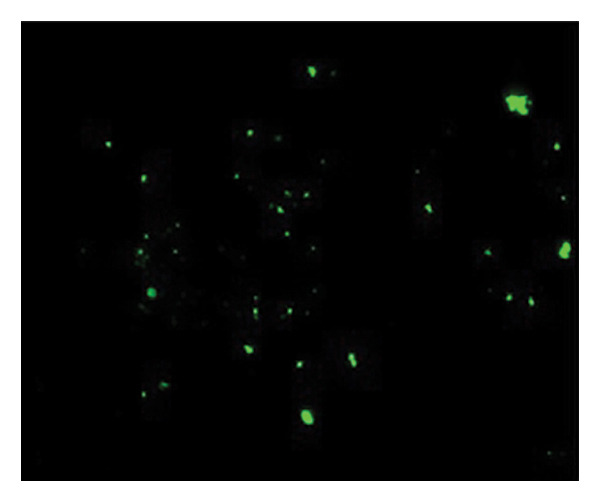
(e)

**Figure:**
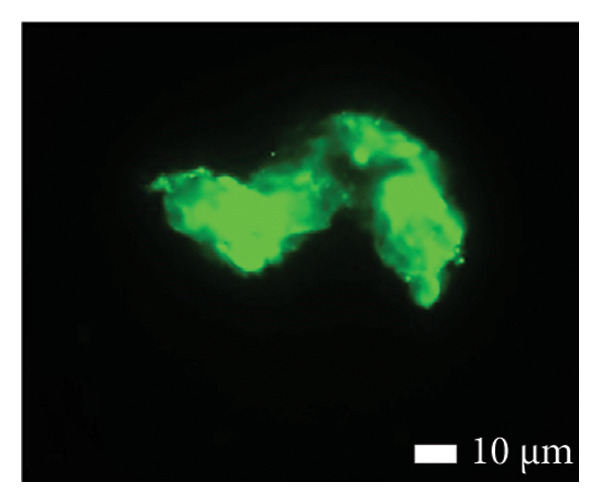
(f)

## 6. Discussion

We investigated the effect of DDAVP, at a clinically applicable concentration, on platelet function without the presence of the vascular endothelium. TEG analysis showed a decrease in the K‐time and an increase in the MA. Fluorescence microscopy examining microclot formation (predominantly fibrin and plasma proteins) as well as platelet activation showed significant increases in both parameters in the DDAVP test samples.

In our study, the addition of DDAVP to blood samples, in vitro, did not demonstrate any effect on PFA‐200 aperture closure times in either of the two sets of agonists, with differences between the means of 0.2 and 0.0 s. An in vivo study in 2014 showed that DDAVP shortened both the COL/ADP and COL/EPI PFA‐200 aperture closure times by 40 s (*p* < 0.001) and 68 s (*p* < 0.001), respectively [2]. The presence or absence of the endothelium was not the only notable difference between these studies as the population included in Colucci et al.’s study recruited patients with primary platelet function defects [2]. The addition of DDAVP may have been able to return platelet function to within normal clotting parameters in the subjects with pathology due to a transient rise in vWF antigen (vWF:Ag); however in our study, no effect was observed possibly because platelet stores of vWF:Ag are considerably less than those supplied in the endothelium and therefore DDAVP could not promote any change in platelet functioning.

Previous studies utilizing VET testing have not shown any effect [18]. TEG K‐time represents the time from a clot amplitude of 2 mm to progress to 20 mm, while the TEG MA represents the maximal strength of the thrombus. K‐time and MA are both subject to fibrinogen concentration, platelet count, and platelet function [19]. The prothrombotic changes observed in the TEG K‐time and MA DDAVP samples are congruent with one another and suggest that either one or more of the above parameters affecting them have been enhanced. Since these outputs are a composite of interacting blood constituents, it is not possible from this analysis alone to identify which parameter/s have been altered.

Findings from the fluorescence microscopy examining microclot formation offer a reasonable explanation for the prothrombotic findings in the TEG data [6, 7]. From this, we could infer that the K‐time and MA were potentially influenced by both an increase in fibrin formation as well as enhanced platelet function. It is not possible, however, to determine if one effect was more prominent than the other or if one effect was solely responsible for the witnessed changes and the other was observed by chance due to an unknown confounder. To our knowledge, this is the first study to demonstrate this effect.

While PPP is generally considered devoid of intact cells, following centrifugation at 3000 × g for 15 min, it is important to acknowledge that small amounts of cellular debris, including from platelets or circulating endothelial cells, may remain and become entrapped within fibrinaloid microclot complexes. These structures are prominently stained by ThT, indicating a high amyloid content consistent with plasma protein misfolding. We therefore interpret the larger structures seen in Figure 3 not as intact cells but as aggregates of misfolded fibrin (ogen) and other plasma proteins, potentially incorporating residual membrane fragments. The presence of circulating endothelial‐derived material cannot be excluded and may reflect a prothrombotic response to DDAVP independent of vWF release, as supported by previous studies showing DDAVP‐induced tissue factor exposure by endothelial cells [20]. Further studies employing specific markers for endothelial cells could help clarify this possibility.

The results of this study, although positive, should be interpreted with the knowledge that the landscape of platelet function testing to date has not provided any definitive answers with regards to whether DDAVP has a direct effect on platelet function or not. This may seem intuitive; however, it is noted that this question has been studied extensively with many studies supporting and refuting the conclusion that DDAVP has a direct effect on platelet function. Heterogeneity, with regard to methods of platelet function analysis, timing of testing, dosing of DDAVP, testing in vitro versus in vivo, and population selection, likely explains these contrasting results [6–8, 18, 21].

Study limitations include convenience sampling and the fact that none of the investigators were blinded as to which were test and control samples. The area potentially most affected by this is the fluorescence microscopy data, as this lends itself to confirmation and observer bias by those examining the slides. The addition of flow cytometry would provide a more robust quantitative measure of platelet activation and limit the influence of observer bias. [22] It should be noted, that the study was restricted to healthy adults aged 18–50 years of age and therefore the results cannot be extrapolated clinically to patients at the extremes of age or those with an underlying blood dyscrasia.

## 7. Conclusion and Recommendations

This is the first study, to our knowledge, demonstrating a positive in vitro effect of DDAVP on prothrombotic parameters examined by VET testing and fluorescence microscopy. A relationship exists between DDAVP exposure in vitro and potentiation of clot formation. Fluorescence microscopy confirms that DDAVP activates platelets without mechanisms mediated by the endothelium. This presents an opportunity to improve on this study by a more rigorous interrogation of the in vitro effect of DDAVP on platelet function and the prothrombotic cascade, as this may be important for future pharmacological manipulation. Future studies would benefit from a larger study sample and double blinding the investigators. Additional testing in the form of whole blood impedance aggregometry, light transmission aggregometry and flow cytometry would offer more specific insights into the effect of DDAVP on platelets in vitro. Population sampling should be randomized, and the inclusion of a broader demographic, including participants who are pregnant and the pediatric population, would help further clarify DDAVP’s interaction with platelets and plasma proteins.

## Ethics Statement

Ethical approval for the study was granted by the Human Research Ethics Committee, Stellenbosch University, South Africa (Reference number: S22/08/140).

## Consent

Informed consent was obtained from all participants.

## Disclosure

All the authors approved the final version of the manuscript.

## Conflicts of Interest

The authors declare no conflicts of interest.

## Author Contributions

Michael V. Forner, Sean Chetty, and Etheresia Pretorius designed the study protocol.

Michael V. Forner recruited study participants, gained informed consent, and collected blood samples for the study.

Michael V. Forner performed PFA‐200 and TEG analysis of the blood samples.

Etheresia Pretorius and Chantelle Venter performed platelet microscopy and microclotting analysis.

Michael V. Forner, Sean Chetty, Etheresia Pretorius, and Chantelle Venter carried out statistical analysis and interpretation of the data.

Michael V. Forner wrote the manuscript.

Sean Chetty, Etheresia Pretorius, and Chantelle Venter critically reviewed the manuscript.

## Funding

This research was supported by The Harry Crossley Foundation (Stellenbosch University), the Jan Pretorius Research Fund (South African Society of Anesthesiologists), and the Critical Care Society of Southern Africa.

## Data Availability

Data are available on reasonable request from the authors.
